# Complete Goss Secondary Recrystallization by Control of the Grain Size and Texture of Primary Recrystallization in Grain-Oriented Silicon Steel

**DOI:** 10.3390/ma14185383

**Published:** 2021-09-17

**Authors:** Zhanyi Xu, Yuhui Sha, Zhenghua He, Fang Zhang, Wei Liu, Huabing Zhang, Liang Zuo

**Affiliations:** 1 Key Laboratory for Anisotropy and Texture of Materials, Ministry of Education, Northeastern University, Shenyang 110819, China; mythxzy@163.com (Z.X.); zhangf@smm.neu.edu.cn (F.Z.); lvv0722@126.com (W.L.); lzuo@mail.neu.edu.cn (L.Z.); 2 School of Materials Science and Engineering, Shenyang University of Technology, Shenyang 110870, China; hezhenghua@mail.neu.edu.cn; 3 Baoshan Iron & Steel Cooperation Limited, Shanghai 201900, China; 710994@baosteel.com

**Keywords:** Hi-B steel, abnormal grain growth, primary recrystallization, grain size, texture, island grain

## Abstract

Matrix microstructure and texture controlling is an important way to optimize Goss ({110}<001>) abnormal grain growth (AGG) in high magnetic induction grain-oriented silicon (Hi-B) steel during primary recrystallization. In the present work, a matrix with homogeneous grain size and favorable texture components was obtained through two-stage normalized annealing followed by primary recrystallization. Furthermore, secondary recrystallization was performed for sharp Goss orientation by slow heating and purified annealing. It was found that plenty of island grains, which occurred and disappeared gradually, accompanied the process of AGG. Through analyzing the evolution of microstructure and texture, we realized that the formation of island grains was related to the large-size grains in matrix, and the elimination of that was attributed to the special grain boundaries which satisfied both coincident site lattice (CSL) and high-energy (HE) models. It was essential to control grain size and favorable orientations in matrix comprehensively for the high-efficient abnormal growing of sharp Goss orientation, through which excellent magnetic properties could be obtained simultaneously.

## 1. Introduction

Grain-oriented silicon steel is the basic material for manufacturing transmission and distribution transformer cores. It is known for its long manufacturing process, which is complex and highly technical. To improve the magnetic properties, mechanism and high-efficacy control of secondary recrystallization (also known as abnormal grain growth, AGG) of sharp Goss ({110}<001>) grains have always been the major issues in this field [1,2,3]. Although an increasing amount of attention has been devoted over the last decades, it is still the subject of ongoing interest.

The behavior of Goss AGG has strong correlations with the matrix of primary recrystallization in grain-oriented silicon steel. Microstructure [4,5,6], texture [7,8,9], and inhibitors [10,11] in the matrix all play important roles in this process. The homogeneous primary recrystallization microstructure under the restriction of inhibitors is beneficial to maintain the stability of matrix during high-temperature annealing [11,12,13]. It is also favorable to keep relative large-size grains in matrix within a reasonable proportion for the highly efficient execution of sharp Goss AGG [14,15]. Meanwhile, increasing favorable texture components in primary recrystallization also promotes this process. For example, {114}<481> and {111}<112> both satisfy the coincident site lattice (CSL, Σ9 grain boundary) and high-energy (HE) (grain boundary misorientation of 20–45°) models with Goss orientation, which is advantageous to the AGG of sharp Goss orientation [16, 17, 18, 19]. Especially for high magnetic induction grain-oriented silicon steel (known as Hi-B steel), these texture components should be enhanced for the high mobility of Σ9 grain boundary [20].

During the long preparation process of Hi-B steel, the coordinated and stable control of multi-factors is extremely complicated. Grain size of the primary recrystallization matrix, which is a critical factor for the control of AGG, usually presents a discrete distribution state. Grains of {114}<481> are easy to obtain large sizes in the colony due to their recrystallization behaviors [19,21]. Furthermore, Liu et al. [22,23] found that peninsula or island grains will also form in these textures. Furthermore, if such a phenomenon is not restricted, non-Goss orientations, such as {210}<001> and {110}<112>, could get the opportunity to grow and form island grains which are difficult to eliminate in the final products [23,24]. These island grains will interfere with the process of Goss AGG and deteriorate the magnetic properties of final products [24].

Related researches [14,15,25,26,27] show that the formation of peninsula or island grains is directly related to the factors of grain boundary characteristics and grain size during AGG. Bennett et al. [25] pointed out that matrix grains with Σ3 boundaries could act as pinning points during AGG because of the low mobility, and island grains may form for such a reason. Rajmohan, Shin et al. [26,27] found that peninsula or island grains around and in abnormally growing Goss grains were mainly composed of either low- (<15°) or high- (>45°) angle grain boundaries. Etter, Mazzi et al. [14,15] considered that the grain boundaries with misorientation of 20–45° do not have special mobility, and the large-size matrix grains are easier to form island grains.

Based on the above results, we can see that the unfavorable texture components and discrete grain size distribution in the primary recrystallization matrix both restrict AGG significantly. For Hi-B steel, due to the strong genetic effects in microstructure and texture [28,29], the adjustment of initial matrix before cold rolling is an important way to control the primary recrystallization matrix. Relevant researches have shown that the normalized annealing increases the proportion of favorable texture components [30,31], and that the average grain size and the uniformity of microstructure are also improved [32,33]. However, the microstructure obtained by conventional normalized annealing still retains a relatively larger state in the center area, the problems of microstructure are different in different layers, and the discrete grain size distribution of special orientations have not been solved effectively.

In this study, to obtain a more favorable initial matrix and further explore the evolution mechanism of island grains, the special two-stage normalized annealing was combined to achieve targeted regulation. The primary recrystallization matrix with homogeneous grain size and favorable texture components was obtained, which led to stable and highly efficient AGG of sharp Goss orientation accordingly. The evolution of microstructure and texture was investigated to clarify the formation and elimination of island grains. Meanwhile, based on experimental and model results, the comprehensive control strategy for AGG of Goss orientation in Hi-B steel is also proposed, which is under the coupling effects of microstructure, texture, and inhibitor of the primary recrystallization matrix. It is of great significance for the pursuit of extreme magnetic properties of advanced Hi-B steel.

## 2. Materials and Methods

**Figure 1:**
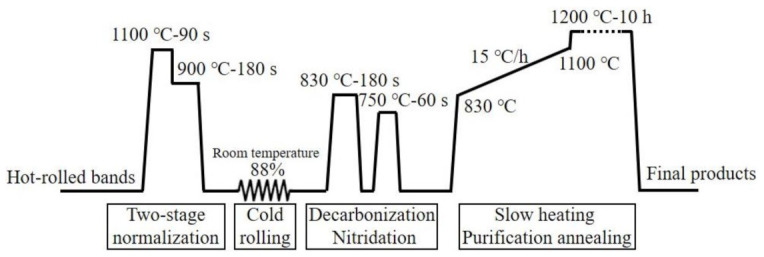
Schematic diagram of the experimental procedure.

Industrial Fe-3.25 wt.%Si Hi-B steel (Baoshan Iron & Steel Cooperation Limited, Shanghai, China) was used as the raw materials in this study, in which AlN and MnS were taken as the main inhibitors. The initial slabs were heated below 1200 °C and hot-rolled into the bands of 2.3 mm, and the finishing rolling temperature was about 900 °C. After treating the surface of hot-rolled bands at room temperature, two-stage normalization was performed, that is, annealing the hot-rolled bands at 1100 °C for 90 s and then holding at 900 °C for 180 s. After cold rolling with a reduced rate of 88% at room temperature, the strips were annealed at 830 °C for 3 min in a wet atmosphere comprising 60% H_2_ and 40% N_2_ for decarbonization and primary recrystallization. Subsequently, the strips were nitrided in a gas mixture containing NH_3_, H_2_, and N_2_. Then, the strips were slowly heated from 830 °C to 1100 °C at a rate of 15 °C/h in a dry atmosphere of 75% H_2_ and 25% N_2_. Finally, the high-temperature purification annealing was carried out at 1200 °C for 10 h with the atmosphere of pure H_2_. A schematic diagram of the experimental procedure is shown in Figure 1.

The secondary recrystallization starting temperature (T_AG_) of the strips is determined by interrupted annealing. Samples were taken out at every 20 °C from 980 °C to 1060 °C during the process of slow heating annealing. The evolution of island grains in the annealing temperature range was then analyzed at the same time. Besides, quasi-in-situ annealing was carried out at T_AG_ in a vacuum furnace (Kejing, Hefei, China) to capture the evolution of microstructure and texture during AGG of Goss orientation.

**Figure 2:**
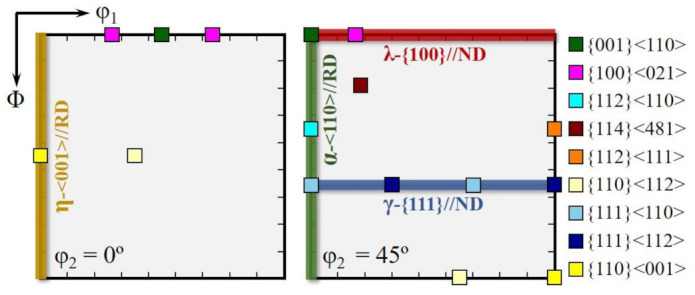
Constant φ_2_ = 0° and 45° sections of ODFs showing some important texture components and fibers of bcc metals in Bunge system.

Magnetic properties were measured using a single sheet tester (IWATSU, Tokyo, Japan), including B800 (magnetic flux density at 800 A/m) and P17/50 (core loss at 50 Hz and 1.7 T) of the strips after the purification annealing. At different stages in the preparation process, the micro-texture analysis was carried out by EBSD on a JEOL JSM-7001F scanning electron microscope (Tokyo, Japan) operating at an acceleration voltage of 20 kV and a working distance of 15 mm. Observation of inhibitors was also carried out using the same equipment. All micro-texture and grain size measurements were conducted using HKL Technology Channel 5 software (5.0.9.0, HKL Technology A/S, CT, U.S.). The tolerance angle of 15° was adopted to define a texture component. The orientation distribution function (ODF), based on the data of EBSD, is represented by Bunge notation [34]. The positions of the main texture components in this work are shown in Figure 2.

## 3. Results

### 3.1. Microstructure, Texture and Inhibitor in the Rolling and Primary Recrystallization Strips

**Figure 3:**
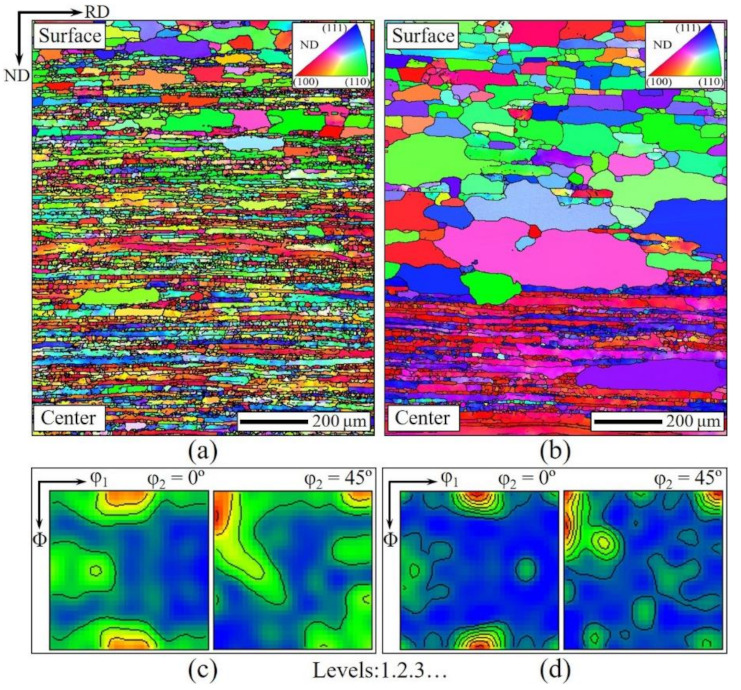
Microstructures (**a,b**) as well as constant φ_2_ = 0° and φ_2_ = 45° sections of ODFs (**c,d**) of hot-rolled and normalized bands.

Figure 3 shows the microstructure and texture of hot-rolled and normalized Fe-Si bands. The hot-rolled band (Figure 3a) exhibited elongated grains along the rolling direction through thickness. The small-size grains distributed in the regions of grain boundary are formed by phase transformation and dynamic recrystallization during hot-rolling [35,36]. The hot-rolled texture (Figure 3c) is characteristic of strong α-fiber (<110>//RD), weak γ-fiber ({111}//ND) and shear texture including Goss, {110}<112>, and {112}<111>. Due to the high finishing temperature of the hot-rolling process, the temperature of normalized annealing also needed to be high. Thus, if the conventional normalized annealing is used, the long-term high-temperature annealing treatment will cause significant recrystallization in different areas of the matrix, and the central area will also obtain large grains, which is undesirable.

It is reported that the shear strain is weakened and the temperature reduction rate is gradually slow from the surface to the central area of the bands during hot rolling [28]. This results in a decreasing distribution of energy storage from the surface to the center in the hot-rolled bands. This gradient distribution of energy storage provides the possibility of differential control of recrystallization. Promoting recrystallization and grain growth from the surface to the sub-surface area, as well as inhibiting recrystallization in the central area and keeping it in the hot-rolled state, not only helped to avoid the appearance of large-size grains in the center, but also proved to be beneficial to the subsequent recrystallization texture control. Considering the finishing temperature of the hot rolling process and the gradient distribution of energy storage in the hot-rolled bands, the differential control of recrystallized behavior in different layers needed to be combined with annealing treatment at different temperatures. The conventional normalized annealing at a single temperature was difficult to achieve that. Therefore, two-stage normalized annealing was used in this study.

For the first-stage high-temperature normalized annealing, a shorter time was required. During the process, full recrystallization happened in the surface and sub-surface area with high energy storage, while the central area with relatively lower energy storage could only recover or partially recrystallize [37]. The second-stage low-temperature annealing with a relatively long time promoted the complete growth of recrystallized grains in the surface area and accelerated the full release of stored energy in the center. Thus, the recrystallization in different regions of the bands showed different results after the two-stage normalized annealing, as shown in Figure 3b. This is distinct from the previous researches on the normalized bands, which are composed of small-size equiaxed grains near the surface area and coarse elongated grains in the central area [32,33]. The texture level of α-fiber improved as a result of strong recovery and partial re-crystallization in the central layer which is the main existing area of α-fiber. Meanwhile, the weakening of Goss is the result of obvious recrystallization and grain growth of the subsurface layer.

**Figure 4:**
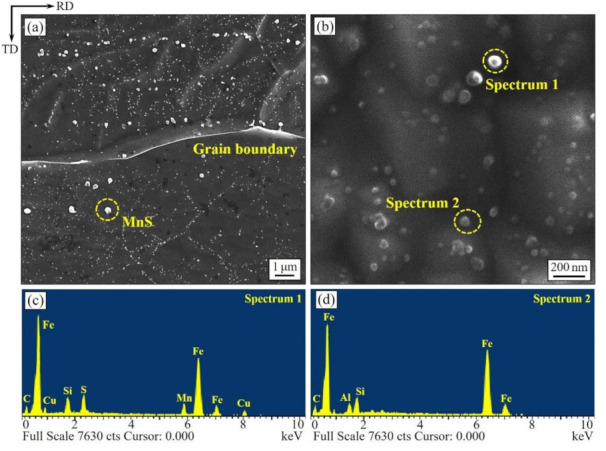
SEM images (**a,b**) and EDS analysis (**c,d**) of the precipitates in the matrix after normalized annealing.

Figure 4 presents the characteristics of precipitations in the normalized bands. Large-number and high-density precipitates inside grains are observed after annealing. The precipitates with a size larger than about 70 nm are mostly identified as MnS, and the smaller ones with a size of about 20 nm are identified as AlN by EDS. Such results indicate that the two-stage normalized annealing also promotes precipitation inhibitors. The diffuse and finely distributed inhibitors, on the one hand, help to inhibit the growth of grains during primary recrystallization and ensure the homogeneous of microstructure; on the other hand, they can inhibit the growth of matrix grains during the slow heating annealing and can ensure the stability of matrix. This provides a guarantee for the highly efficient and stable occurrence of secondary recrystallization [2].

**Figure 5:**
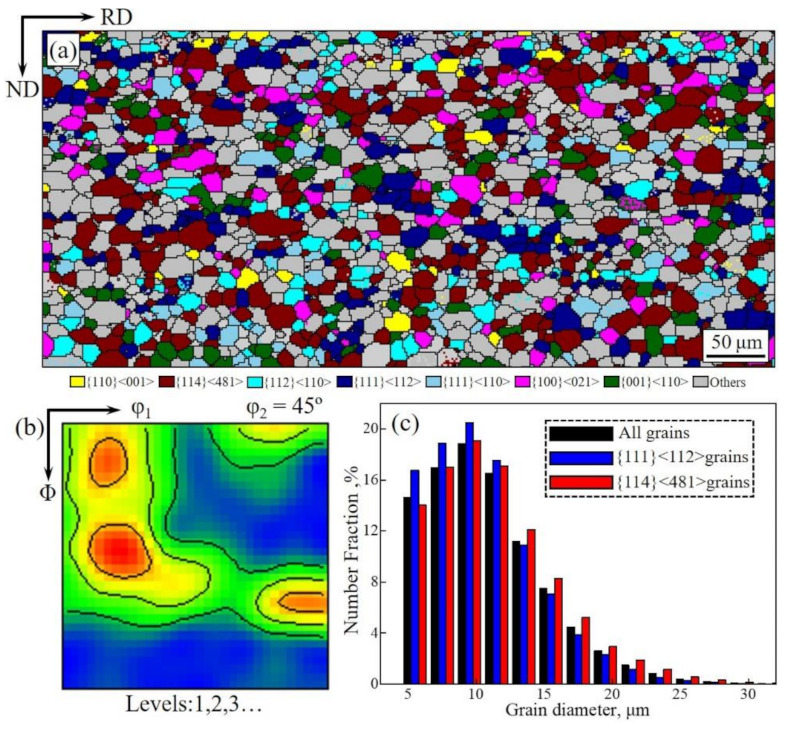
Orientation image map of main texture components (**a**), constant φ_2_ = 45° section of ODFs (**b**) and grain diameter distribution of all grains, {111}<112> and {114}<481> grains (**c**) in the Fe-Si strips after primary recrystallized annealing.

Figure 5 shows the microstructure and texture of the Fe-Si strips after primary recrystallized annealing. The microstructure is featured by homogeneous grains with an average size of about 10 μm. The texture is mainly composed of {114}<481> and γ-fiber texture with the peak at {111}<112>. The area fractions of {114}<481> and {111}<112> textures are 19.4% and 15.8%, respectively. According to statistics, {111}<112> grains with an average size of about 9.5 μm exhibit a relatively lower distribution than {114}<481> grains with an average size of about 11 μm, but the grain size distributions of different texture components still show similar states. It is noted that the {114}<481> grains are featured by the equiaxed shape and its size distribution is narrower than that reported in references [22].

According to relevant researches, the characteristics of large-size {114}<481> grains in the primary recrystallization matrix are related to their recrystallization behavior in deformed α-fiber grains [19,21,38]. In this work, the initial α-fiber grains in the center area present a small size and high grain boundary ratio, which are obtained through the designed two-stage normalized annealing. This leads to more frequent nucleation of γ-fiber grains at grain boundaries and restricts the growth of {114}<481> grains [39]. Thus, homogeneous microstructures could be obtained after cold rolling and primary recrystallized annealing; the colony of large-size {114}<481> grains could also be avoided simultaneously.

### 3.2. Evolution of Microstructure and Properties during Secondary Recrystallization

**Figure 6:**
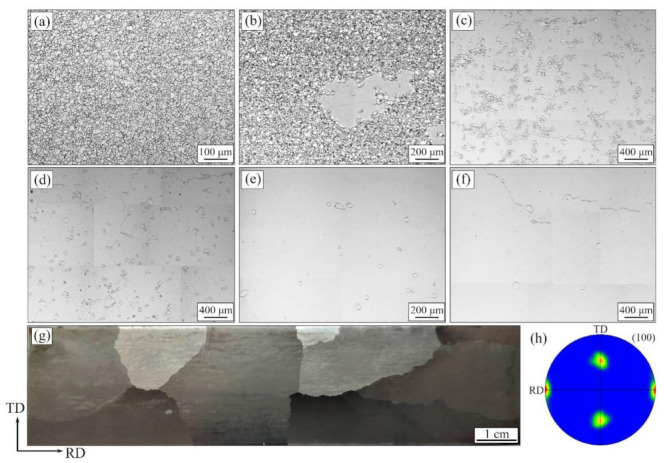
Microstructure evolution during slow heating annealing of the Fe-Si strips as heated to 980 °C (**a**), 1000 °C (**b**), 1020 °C (**c**), 1040 °C (**d**), 1060 °C (**e**), and 1080 °C (**f**). In addition, macrostructure (**g**) and (100) pole figure (**h**) of final product after purification annealing.

Figure 6 presents the microstructure evolution of the Fe-Si strips during slow heating annealing from 980 °C to 1080 °C, as well as the macrostructure and texture of the final products after purification annealing. The uniform and stable microstructure were observed at 980 °C with an average grain size of 12 μm. This is attributed to the control of fine and dispersion inhibitors. As annealing temperature arises to 1000 °C, the abnormally grown grain with a size of about 400 μm appeared, and T_AG_ of the researched the Fe-Si strips is approximately considered to be 1000 °C. As annealing temperatures raised to 1020 °C, widespread AGG was observed. Meanwhile, lots of island grains distributed dispersedly in the abnormal growing grains were consumed rapidly as annealing temperature increased to 1040 °C. Further, these island grains almost disappeared when they were heated at 1060–1080 °C. After the final purification annealing, the product presented a perfect secondary recrystallization of sharp Goss at an average grain size of 2–3 cm with no island grain remained.

Table 1 shows the evolution of density and an average size of the island grains during the slow heating annealing. The density decreased significantly as annealing temperature increased, and the average size of these grains increased. The grain size of island grains was much smaller than that in the relevant reports as annealing temperature higher than 1040 °C [24]. This means that the treatment in this study restricts the development of island grains and provides a certain condition for the complete consumption of these grains after the final purification annealing.

**Figure 7:**
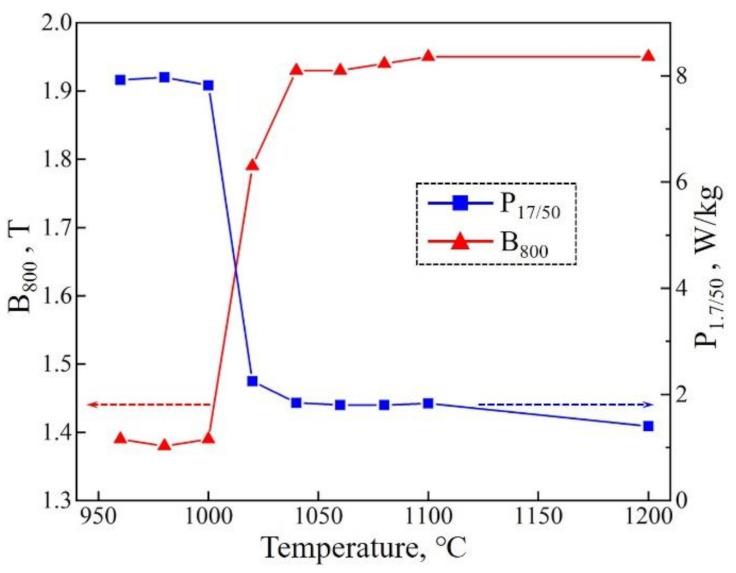
Change of B_800_ and P_1.7/50_ during slow heating annealing and after the final purification annealing.

The magnetic properties of the Fe-Si strips during slow heating annealing are shown in Figure 7. The widespread occurrence of Goss AGG near 1020 °C leads to evident improvement of B_800_ and P_1.7/50_. The rapid elimination of high-density island grains as annealing temperature exceeds 1020 °C also improves the magnetic properties. It is noted that the properties are almost stable at the temperature range of 1040–1100 °C. Only after the final purification annealing at 1200 °C does P_1.7/50_ further decrease due to the elimination of residual island grains and the elements which is harmful to magnetic properties. The B_800_ finally reaches 1.95 T, and P_1.7/50_ decreases to 1.3 W/kg.

The behavior of AGG and magnetic properties as obtained show that the AGG of sharp Goss orientation could proceed at a high growth rate and be completed in a short time or temperature range (1000–1040 °C). This is attributed to the favorable matrix of primary recrystallization obtained by the targeted treatment of two-stage normalized annealing. At the same time, it is possible to shorten the slow heating annealing process and the product preparation cycle of Hi-B steel. Moreover, the appearance and elimination of a large number of island grains during AGG are also meaningful, because they would interfere with the stable and high-efficient control of secondary recrystallization process. Therefore, such a phenomenon will be analyzed and discussed in the following.

## 4. Discussion

### 4.1. Formation and Evolution Mechanism of the Island Grains

**Figure 8:**
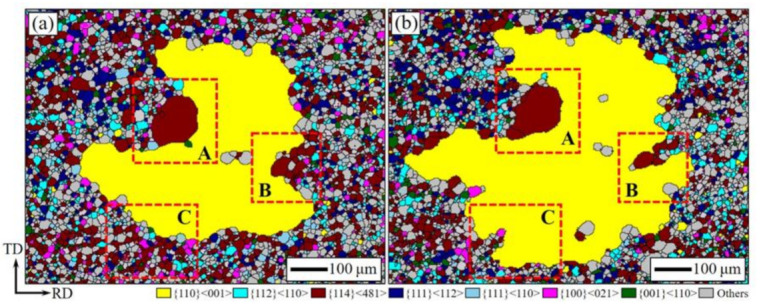
Quasi-in-situ EBSD analysis of the Fe-Si strips: heating to T_AG_ during slow heating annealing (**a**); after annealing at T_AG_ for 100 s (**b**).

Island grains influence the process of AGG, and the residual island grains are detrimental to the magnetic properties of grain-oriented silicon steel [23,24]. It is required to reveal the formation mechanism of these grains. Quasi-in-situ annealing and EBSD analysis were carried out to analyze the evolution of AGG accurately, as shown in Figure 8. The annealed strip containing AGG of Goss was held at T_AG_ for 100 s. Comparing the microstructure before and after the treatment, it is found that the abnormally growing Goss grain shows apparent shape anisotropy. As shown in the marked region A and B, a strong pinning effect, developed by the large-size {114}<481> peninsula grains, makes it difficult for the growing process to proceed in those directions. The Goss grain tends to bypass the pinning points and form island grains inside. The pinning effect of large-size {114}<481> grains is mainly formed by some individual large-size matrix grains.

**Figure 9:**
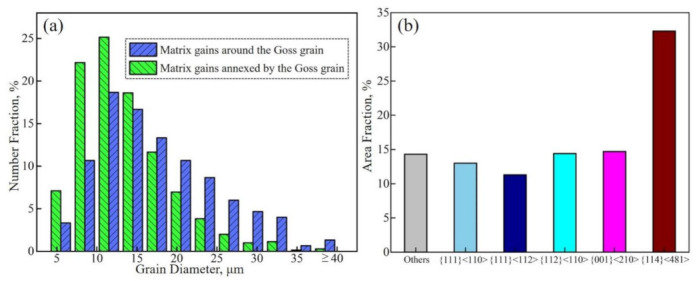
Grain diameter distributions of the matrix grains around the abnormally growing Goss grain before the quasi-in-situ annealing and the matrix grains consumed during the process (**a**), as well as the area fraction of main texture components of the consumed matrix grains (**b**).

Grain size distributions of the matrix grains around the abnormally growing Goss grain before the quasi-in-situ annealing and the consumed matrix grains during the process are presented in Figure 9a. It appears that the consumed matrix grains are mostly small-sized. The relatively larger ones are difficult to consume rapidly, and will be present at higher temperatures and form peninsula or island grains. The texture composition of the consumed region is shown in Figure 9b. The area fractions of {114}<481> and {111}<112> grains in this region are 33% and 11%, respectively. This means the fraction of favorable texture components, which satisfy both CSL (Σ9) and HE (20–45° misorientation) models with Goss, account for up to 44% in this region. Furthermore, most of them are in a small-size size, as seen in Figure 9a. In other words, the matrix grains with favorable texture components can promote the AGG of Goss, but some larger ones will still interfere with this process and form peninsula or island grains.

**Figure 10:**
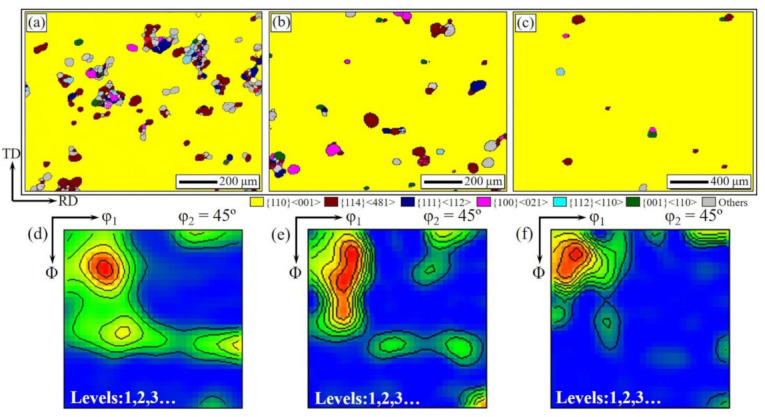
Orientation image maps of the Fe-Si strips (**a**–**c**) and constant φ_2_ = 45° section of ODFs of the island grains (**d**–**f**) during the slow heating annealing as heated to 1020 °C (**a**,**d**), 1040 °C (**b**,**e**), 1060 °C (**c**,**f**).

For analyzing the island grains in more detail, orientation image maps of the Fe-Si strips and constant φ_2_ = 45° section of ODFs of the island grains during the slow heating annealing are shown in Figure 10. The island grains inside the abnormally growing Goss grains present multi-component textures. It is noted that the strong γ-fiber texture at 1020 °C is gradually eliminated with the increase in the annealing temperature, while {114}<481> texture is always at the highest level throughout the given temperature range, as shown in the ODFs of Figure 10d,e,f.

**Figure 11:**
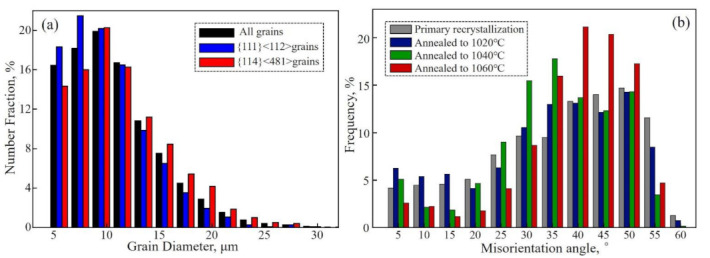
Grain diameter distribution of all grains, {111}<112> and {114}<481> grains in the Fe-Si strips at T_AG_ (**a**). The misorientation angle distribution of Grain boundary in the Fe-Si strips after primary recrystallization and during slow heating annealing as heated to different temperatures (**b**).

According to related researches, the formation mechanism of island grains is mainly related to grain size [14,15] and grain boundaries characteristics [25,26,27]. The grain size distributions of matrix and main texture components at 980 °C are shown in Figure 11a. The maximum size of matrix grains is more than 30 μm. By comparison with the average size of island grains in the formation stage, as given 29.8 μm at 1020 °C in Table 1, it can be inferred that the grain size plays an important role in the formation of island grains. As the main texture components in matrix, {114}<481> and {111}<112> grains also contributed a large number in the large-size range. This makes them form the island grains, especially {114}<481> which occupies the dominant fraction can survive to the later stage of AGG.

The grain boundary misorientation distributions of primary recrystallization matrix and those around the island grains as heating to different temperatures are shown in Figure 11b. The fraction of high-angle grain boundaries, especially the misorientation of 35°~45°, increases significantly with the temperature rising. This is because {114}<481> and {111}<112>, the main textures of island grains, can form orientation relationships of 39.4°<110> and 35.4°<110> with Goss, respectively, which satisfied both CSL and HE models. Such a phenomenon is inconsistent with the studies stating that island grains are easy to form grain boundaries with the characteristics of Σ3, low (<15°) or high (>45°) angle, which are hard to move [25,26,27]. Although several {114}<481> island grains can survive to the later stage of AGG, they are still in a relatively small-size state comparing with the island grains as reported in other researches [23]. Meanwhile, it can be completely eliminated by the advantage of the special grain boundaries after the high-temperature annealing. This is attributed to the two-stage normalized annealing with the aim of controlling the initial state, which provided a favorable microstructure and texture environment for the primary recrystallization matrix.

### 4.2. AGG of Goss under the Influence of Multi-Factors

Influence of various sensitive factors during the AGG can be analyzed from the perspective of model. Based on the Hillert kinetics model of grain growth [4], Ushigami et al. [11,12,13] characterized the combined action of grain size, texture, and inhibitor during AGG, as expressed by Equation (1):

(1) $$\frac{{\mathrm{dR}}_{G}}{\mathrm{dt}}{\;=\;\alpha m\gamma }_{m}\left(\frac{1}{R_{m}}\;-\;\frac{e}{R_{G}}\;-\;\frac{\mathrm{eZ}}{\alpha }\right)$$

where R_G_ is the equivalent radius of the Goss grain; R_m_ is the radius of matrix grains; α is the geometrical factor; t is time; m is the grain boundary mobility; γ_m_ is the mean grain boundary energy of the matrix; Z represents the Zener inhibitor factor; and e is the relative grain boundary energy coefficient, defined as e = γ_g_/γ_m_ [11,12,13]. Furthermore, γ_g_ represents the grain boundary energy between Goss and the matrix grains that can form favorable orientation relationships.

Based on the above model, it can be inferred that matrix grains with random textures appear as e = 1 during the AGG of Goss [11,12,13]. In addition, the grains of favorable textures, such as {114}<481> and {111}<112>, will present e < 1 with Goss and provide more driving force for the growing process [11,12,13]. However, such an advantage is carried out under the significant influence of the matrix grain size, the R_m_ in Equation (1). The small-size matrix grains provide a larger driving force for AGG, and the consuming process can be faster. For the large-size ones, this process is difficult to carry out. As shown in the marked region A and B in Figure 8, although the {114}<481> grains had the advantages of grain boundary characteristics, they were still difficult to consume due to their large size, which led to the pinning effect. AGG in these two directions is blocked, while growth in the other directions is still ongoing. As shown in the microstructure of small-size {114}<481> grains in region C, the AGG process is rapid. The different growth rate of AGG in different directions leads to the shape anisotropy. For the matrix with a larger number of large-size grains, the pinning effect will also increase accordingly, which will further interfere with AGG. Based on the analysis of the model and experiments, it is very important to control the rationality of grain size distribution while ensuring the matrix texture is in a favorable state. That is, the stable and highly efficient AGG is the result of the combined effect of matrix texture and grain size.

At present, the production of Hi-B steel using low-temperature slab heating technology has become the main trend in the development of this industry. The implementation of new technology can easily lead to a discrete distribution of the initial microstructure. If such a phenomenon is not restricted, the matrix of the primary recrystallization will further deteriorate, making the control of AGG more difficult, and the magnetic properties of the products will also be affected. However, the conventional normalized annealing reported in the researches cannot solve this problem well [30,31,32,33]. In this study, based on the differential control of the recrystallization behavior of the hot-rolled bands, two-stage normalized annealing was proposed to ensure the homogeneous primary recrystallization matrix. This homogeneous state is not only reflected in the grain size of different layers, but also in the consistency of the grain size distribution among different texture components. This can promote the AGG of Goss at a higher rate, which helps to limit the development of island grains so that no island grains remain in the final product. This not only provides a path for the development of Hi-B steel with more favorable magnetic properties but also provides the possibility to shorten the product process, which is of great significance in both theory and practical application.

## 5. Conclusions

1. The present work produced Fe-3.25 wt.% Si strips with more favorable magnetic properties successfully. After the two-stage normalized annealing, the grain size in different layers of the primary recrystallized matrix was more homogeneous, and the grain size distribution of the main texture components, i.e., {111}<112> and {114}<481>, also showed a similar state. This not only enabled the secondary recrystallization of Goss-oriented grains to be completed efficiently, but also facilitates the complete elimination of island grains, leaving no island grains in the final products.
2. The mechanisms of formation and evolution for island grains during the AGG of Goss are investigated. It is concluded that the main reason for the formation of island grains is related to the size of matrix grains, and the elimination of these grains is attributed to the characteristics of grain boundaries.
3. A comprehensive analysis of Goss AGG is carried out under the influence of grain size and texture in the primary recrystallization matrix. It is considered that the effect of favorable texture components needs to be coordinated and controlled by the factor of grain size. In this way, the stable and highly efficient control of secondary recrystallization of sharp Goss oriented-grains can be realized.

## Figures and Tables

**Table 1:** Density and average size of island grains in the Fe-Si strips at different temperatures during slow heating annealing.

Temperature (°C)	1020	1040	1060	1080
**Density of Island Grains (/mm^2^)**	280.0	34.5	2.6	0.4
**Average Diameter of Island Grains (μm)**	29.8	42.3	63.2	92.3

## Data Availability

Data is contained within the article.
